# Gait, skin and coat, and plasma cytokine changes in response to exercise and trace mineral source

**DOI:** 10.1093/jas/skaf361

**Published:** 2025-10-22

**Authors:** Claire L Timlin, Sarah M Dickerson, Fiona Mccracken, Patrick M Skaggs, Jason W Fowler, Laura A Amundson, Allison A Millican, Alyssa S Cornelison, Craig N Coon

**Affiliations:** Four Rivers Kennel, Walker, MO 64778; Four Rivers Kennel, Walker, MO 64778; Four Rivers Kennel, Walker, MO 64778; Four Rivers Kennel, Walker, MO 64778; Four Rivers Kennel, Walker, MO 64778; Zinpro, Eden Prairie, MN 55344; Zinpro, Eden Prairie, MN 55344; Zinpro, Eden Prairie, MN 55344; Four Rivers Kennel, Walker, MO 64778

**Keywords:** complexed minerals, cytokines, dog, mobility

## Abstract

This study explored whether trace mineral source (Zn, Mn, Cu, Fe, and Se) in a complete and balanced diet impacts gait and inflammation in Labrador Retrievers undergoing a 9-wk exercise regimen. Forty healthy, adult dogs, averaging 3 yr of age, were assigned to diets containing ether inorganic minerals (ING, *n* = 20) or amino acid complexed minerals (TMC, *n* = 20). After a 2-wk dietary acclimation, dogs began a progressively demanding exercise regimen, ending with an 8 km run. Fecal scores and food intake were recorded daily, while body weights and body condition scores were recorded weekly. Skin and coat condition were assessed at baseline, mid-study, and study end. Mobility and pain were evaluated before and after the first and last run using the Liverpool Osteoarthritis in Dogs (LOAD) and Canine Brief Pain Inventory (CBPI) questionnaires. Gait was assessed using the Gait4Dogs walkway system and the FRK Total Gait Inflammation Index Score. Both groups experienced a slight reduction in body weights (*P *< 0.01) and body condition scores (*P *< 0.01), likely due to the added energy expenditure of exercise. Skin and coat condition did not differ between the diets (*P *≤ 0.15). The LOAD questionnaire tended to reveal perceptions of improved mobility after exercise (*P *≤ 0.07), and a perceived improvement in the quality of life for TMC dogs after the final run was detected by the CBPI questionnaire (*P *< 0.01). Gait analysis revealed better left:right (L:R) symmetry ratios for step time (*P *= 0.01) in TMC fed dogs. Dogs from ING group had better cycle time L:R ratio (*P *= 0.03), though ratios for both groups were extremely close to 1:1. A treatment × time interaction for mean pressure L:R ratio revealed TMC dogs had a better ratio after the final run compared to ING dogs (*P *= 0.02), suggesting differences in how the two mineral sources influenced gait dynamics during the exercise regimen. Blood samples collected at 1, 6, and 24 h after the first and last runs revealed changes in several cytokines and chemokines, but only monocyte chemoattractant protein-1 (MCP-1) was influenced by a treatment × time interaction (*P *< 0.01). Circulating MCP-1 increased after the final exercise in dogs fed TMC but not in those fed ING. Overall, the results suggest that amino acid complexed minerals may offer advantages in perceived mobility, some gait parameters, and inflammatory responses during a sustained exercise regimen compared to inorganic minerals.

## Teaser

Diets containing amino acid complexed trace minerals promoted an increase in monocyte chemoattracted protein-1, minor beneficial changes in gait, and improvements to perceived mobility after exercise challenge in healthy dogs when compared to inorganic trace minerals.

## Introduction

The role of trace mineral nutrition on animal production has long been a focus in the livestock industry. However, there is less known about the role of these micronutrients in the overall wellbeing of companion animals. Trace minerals are provided in the complete and balanced diet in one of two forms, inorganic or organic. Inorganic trace minerals (sulfates, carbonates, chlorides, and oxides) are absorbed differently, are often less bioavailable, and less efficiently utilized compared to organic trace minerals (Parks and Harmston, 1994; Ward et al., 1996; Nollet et al., 2007; Trevizan et al., 2013; Liu et al., 2016). Stability, pH, and bioavailability of each organic trace mineral class should be carefully considered when selecting for food usage due to potential variability caused by the chelating agent (Trevizan et al., 2013; Byrne et al., 2021).

Trace minerals play essential roles in various physiological processes and serve as cofactors and structural components of enzymes. Zinc is essential for hundreds of enzyme systems and proteins that are critical for inflammatory and immune responses (Ogawa et al., 2018). Zinc influences innate and acquired immune function, supports Th1 response, and is crucial to skin and mucosal membrane integrity (Wintergerst et al., 2007). Zinc and copper have strong antioxidant functions and help stabilize proteins to make them less prone to oxidation (Klotz et al., 2003). Additionally, cytosolic copper- and zinc-dependent and mitochondrial manganese-dependent antioxidants, superoxide dismutase 1 and 2, respectively, function to maintain antioxidant balance within the body (Zelko et al., 2002). Iron is also crucial in regulating oxidative stress through its role in hydrogen peroxidase, an enzyme that catalyzes the breakdown of hydrogen peroxide to water and oxygen (Isaac and Dawson, 1999).

Replacing inorganic trace minerals with those from organic sources can improve animal health and performance. Broiler chickens supplemented with complexed trace minerals had increased average daily gains and performance compared to those fed inorganic trace minerals (Ghasemi et al., 2020). Similar effects have been observed in swine, with complexed iron and copper improving growth of weaned pigs (Zhao et al., 2014; Sun et al., 2023). Complexed trace mineral sources can also improve immune function, aiding in the growth and performance of animals. Replacing all or part of inorganic dietary mineral sources with organic chelated forms improved antioxidant capacity in chickens (Ghasemi et al., 2020), pigs (Sun et al., 2023), and cattle (Zhao et al., 2015). Furthermore, chelated mineral supplementation may benefit skeletal muscular health. Previous work in horses suggests that amino acid complexed trace minerals may support joint health by increasing cartilage turnover and quickening aggrecan synthesis after lipopolysaccharide challenge (Millican et al., 2019). More so, chelated trace minerals can increase bone content of phosphorus, zinc, and manganese in chickens (Ghasemi et al., 2020).

Improvements in mobility due to dietary intervention are often more obvious in a more compromised population, such as senior dogs, which have altered mobility present during normal ambulation and activity. Senior dogs (average age 9.16 yr) fed amino acid complexed trace minerals (Zn, Mn, Cu, Fe) had significantly increased amount of active time (FitBark) over a 3 mo period, compared to dogs fed inorganic trace minerals (Amundson et al., 2025). However, early detection of slight deviations in mobility has the potential to allow for earlier intervention and prevention of reduced mobility and longevity as the animal ages.

Despite the extensive research in production animals, research on how organic trace minerals can benefit companion animals is limited. Zinc is more readily absorbed in dogs when administered as an amino acid chelate compared to an oxide (Lowe et al., 1994a) and has been shown to accumulate more readily in the coat (Trevizan et al., 2013). Another study noted tendencies for increased glossiness with an organic zinc source, suggesting potential effects on coat health (Pereira et al., 2020). Pereira et al.’s study (2020) also found an increase in CD4+ T-cells (helper T-cells) which could have implications for altered immune response in dogs provided organic forms of trace minerals. Thus, the objective of this study was to determine if organic trace minerals, in the form of a 1:1 metal to amino acid complex, impart differential effects on mobility, skin and coat quality, and cytokine profiles during exercise in a working line of Labrador Retrievers. The hypothesis was that these complexed minerals would provide better support and recovery for animals undergoing training and produce better quality coats compared to their inorganic counterparts. To test this, young adult dogs of working age were provided diets with similar macronutrient compositions and exposed to a 9-wk exercise regimen.

## Materials and Methods

All experimental procedures were approved by the Institute of Animal Care and Use Committee at Four Rivers Kennel under Protocol FRK-47. All technicians involved with animal handling, data collection, and data analysis were blinded to treatments.

### Animals and housing

Forty healthy, young adult, working Labrador Retrievers (20 male and 20 female) were enrolled in this study, averaging 3.4 ± 1.2 yr of age (range 2.0 to 6.3 yr) and 28.4 ± 3.2 kg body weight at enrollment. All dogs were individually housed in temperature-controlled kennels constructed with galvanized chain-link fencing (1.22 m × 1.83 m × 1.83 m × 1.83 m) with access to outdoor socialization yards for 6–8 h per day, dependent on weather. Dogs always had free access to automatic waterers in both the kennels and the yards. All animals were up to date on vaccinations and received monthly prophylactic heartworm and parasite prevention. Body weights and body condition scores (1–9 scale with 1 representing emaciated and 9 representing obese) were measured weekly on each animal.

### Diet and treatments

Treatment diets were formulated to meet nutrient requirements for adult dogs based on the Association of American Feed Control Officials (AAFCO) recommendations (AAFCO, 2022; Table 1). Dogs were blocked by sex, age, and body weight and randomly assigned to 1 of 2 dietary treatments, differing only in trace mineral source. Dietary treatments consisted of 2 different supplemental trace mineral sources of Zn (100 ppm), Mn (25 ppm), Fe (45 ppm), Cu (7 ppm), and Se (0.35 ppm): inorganic (ZnSO_4_, MnSO_4_, FeSO_4_, CuSO_4_, NaSeO_3_) trace minerals (ING, *n* = 20), or amino acid complexed trace minerals (Zinpro, Eden Prairie, MN; TMC, *n* = 20). These were a 1:1 amino acid: metal ratio with lysine or glutamic acid (Zn, Mn, Cu, Fe) or a blend of amino acids (Se). For the duration of the study, diets were provided at maintenance levels based on previous kennel feeding data. Dogs were given a 14-d acclimation period for their respective treatments before beginning the exercise regimen. One week acclimation was originally planned for, but due to severe weather, the start of the exercise regimen was delayed by 1 wk. Males were offered on average 753 ± 34 g of extruded kibble per day, and females were offered 715 ± 30 g of extruded kibble per day. The diets were weighed out in grams each day and dogs were provided at least 45 min to consume their portions. Any food not consumed was collected and weight of orts were recorded.

**Table 1.** Compositions of treatment diets (as-fed basis)

Treatment	ING	TMC
**Ingredients, %**		
** Chicken meal, reg ash 15%**	31.40	31.40
** Milo**	22.55	22.55
** Corn**	22.54	22.54
** Wheat**	21.88	21.85
** Salt**	0.625	0.625
** Beet Pulp**	0.625	0.625
** Vitamin premix^1^**	0.20	0.20
** Control TM premix^2^**	0.12	0
** TMC TM premix^3^**	0	0.15
** Choline chloride 60%**	0.05	0.05
** Oxygon powder**	0.015	0.015
** Chicken fat^4^**	7	7
**Proximate and Mineral Analysis^5^**		
** Dry matter, %**	92.23	92.08
** Moisture, %**	7.77	7.92
** Crude Protein, %**	27.40	26.90
** Crude Fat, %**	13.00	12.30
** Crude Fiber, %**	0.47	1.44
** Ash, %**	6.13	5.68
** Nitrogen Free Extract, %**	45.23	45.76
** Metabolizable energy, kcal/kg^6^**	3,647.1	3,588.6
** Zinc (Zn), ppm**	156	162
** Manganese (Mn), ppm**	43.2	45.8
** Copper (Cu), ppm**	17.8	17.9
** Iron (Fe), ppm**	143	168
** Selenium (Se), ppm**	0.90	0.88
** Iodine (I), ppm**	2.00	2.98
** Calcium (Ca), %**	1.48	1.22
** Phosphorus (P), %**	0.96	0.85

1 Vitamin A, 17,163,000 IU/kg; vitamin D_3_, 920,000 IU/kg; vitamin E, 79,887 IU/kg; vitamin B_12_, 22 mg/kg; riboflavin, 4,719 mg/kg; pantothenic acid, 12,186 mg/kg; niacin, 64,736 mg/kg; folic acid, 720 mg/kg; pyridoxine, 5,537 mg/kg; thiamine, 14,252 mg/kg; biotin, 70 mg/kg.

2 CaCo_3_, ZnSO_4_ (Zn, 8.50%), FeSo_4_ (Fe, 3.81%), CuSO_4_ (Cu, 0.55%), MnO (Mn, 2.12%), Ca (IO_3_)_2_ (I, 0.10%), NaSeO_3_ (Se, 206 ppm).

3 Zinpro ProPath Zn (Zn, 6.89%), Zinpro ProPath Fe (Fe, 3.22%), Zinpro ProPath Mn (Mn, 1.83%), Zinpro ProPath Cu (Cu, 0.45%), Ca(IO_3_)_2_ (I, 0.08), Zinpro Availa Se (Se, 149 ppm)

4 Added to kibble post-extrusion.

5 Mineral values are total levels in the complete diet.

6 Calculated using the modified Atwater equation (AAFCO, 2022).

### Fecal scores

Fecal scores were recorded daily for the duration of the trial. Kennel technicians were trained and evaluated for proper scoring techniques before beginning the trial to ensure consistency. Feces were scored based on what each dog had produced overnight in individual kennels. If a dog did not produce a fecal sample overnight, the dogs were accompanied outside individually to obtain a score. Feces were scored as follows: 1) very loose or liquid, no form, diarrhea, possibly bloody; 2) mix of formed and unformed, mostly loose; 3) formed feces, but mostly soft; 4) well-formed, drier, but not hard, easy to pick up; 5) formed but hard with little or no moisture.

### Exercise regimen

Beginning on either day 15 for males or day 17 for females, all dogs began an exercise regimen lasting 9 wk. To accommodate for blood collection and gait analysis, the first week of the regimen had one 4.8 km run. Weeks 2 to 7 consisted of twice weekly 4.8 km runs. Week 8 consisted of two 1.6 km runs, and week 9 entailed one 8 km final challenge run. Under the supervision of handlers, dogs ran alongside an all-terrain vehicle for the prescribed distance on the property where they were free to run, play, and swim. Each dog was fitted with two collars: one attached to a global positioning system (Garmin Intl, Olathe, KS), and the other with an attached Actical accelerometer (Starr Life Sciences Corp; Oakmont, PA). The Actical accelerometer has previously been validated in dogs (Hansen et al., 2007; Rowlison de Ortiz et al., 2022). This allowed quantification of an individual dog’s activity, exact distance ran, and average moving speed. Activity per kilometer was calculated by dividing the dog’s total activity by their GPS-determined distance ran. There was an equipment malfunction during run #4, so data from 11 of the females’ collars was missing. All other runs performed as planned.

### Blood collection and analysis

A trained veterinary technician collected blood via jugular venipuncture into vacutainer tubes containing potassium EDTA. No more than 6 mL of blood was collected at each time point to ensure that total draw volume did not exceed 1% of total blood volume over a 24-h period. Baseline blood draws occurred on day 0 of the study. Sequential blood draws were performed at the time of the first (day 14) and last (day 70) runs of the exercise regimen. Samples were collected at least 24 h before exercise (baseline), then at 1, 6, and 22–24 h after completion of the runs. Blood was centrifuged at 1,500 × *g* for 15 min, then plasma was siphoned off and stored in 1.5 mL polypropylene tubes at −80°C until analysis. Cytokines were analyzed by Eve Technologies (Calgary, AB, Canada) using the Canine Cytokine 13-Plex Discovery Assay on the Luminex 200 system (Luminex, Austin, TX, USA) and Eve Technologies’ Canine Cytokine 13-Plex Discovery Assay (MilliporeSigma, Burlington, Massachusetts, USA) according to the manufacturer’s protocol. This assay assessed circulating concentrations of granulocyte-macrophage colony-stimulating-factor (GM-CSF), interferon gamma (IFNγ), interleukin (IL) – 2, IL-6, IL-7, IL-8, IL-10, IL-15, IL-18, interferon γ-induced protein 10 (IP-10), keratinocyte chemotactic-like (KC-like), monocyte chemoattractant protein-1 (MCP-1), and tumor necrosis factor alpha (TNFα). Assay sensitivities of these markers range from 3.2 to 21.7 pg/mL for the 13-plex. Individual analyte sensitivity values are available in the MilliporeSigma MILLIPLEX MAP protocol.

### Gait analysis and FRK index

Gait analysis was performed using the Gait4Dogs system and software (CIR Systems, Inc, Franklin, NJ) which records temporal, spatial, and pressure measurements while the dogs traverse the pressure plate walkway and has been previously validated in dogs (Fahie et al., 2018; Guadalupi et al., 2023). Before the experiment, all dogs were acclimated to walking on the mat for several walks. During the study, measurements were taken before and 28 h after exercise. During each data collection period, each dog was walked on the mat 6 to 12 times to obtain at least 3 valid walks. Valid walks consisted of maintaining a constant speed without stopping or stepping off the mat, minimal head turning, minimal leash pulling, and lack of interference from the handler. All valid walks were comprised of at least 3 gait cycles. Video recordings of each walk were reviewed to verify that the inclusion criteria had been met.

For each parameter assessed by the system (Supplementary Table S1), the measurements were averaged for all 4 paws, the forelimb:hindlimb (F:H) ratio was calculated by dividing the sum of the two forelimb scores by the sum of the two hindlimb scores, and the left:right (L:R) ratio was calculated by dividing the sum of the two left limb scores by the sum of the two right limb scores. The Four Rivers Kennel (FRK) Total Gait Inflammation Index score was also calculated as previously reported (Varney et al., 2022). This provides an overall idea of how close a dog is to the “ideal” gait based on the parameters provided by the Gait4Dogs system, and a lower score is equivalent to a better gait. Briefly, the absolute distance of a dog’s score from the ideal measurement is calculated for the following parameters: gait lameness score—a score generated by the software accounting for weight distribution and reach, ideal score of 100 per limb; percent total pressure index—reflects weight distribution for each limb, ideal measurement of 30 for forelimbs and 20 for hind; step/stride ratio—ratio of step length to stride length and reflects torque around the cervical or lumbar spine, ideal measurement of 50 for each limb; hind reach—reflects the flexion and extension ability of the hip, ideal measurement is ½ the step length of the respective hind limb. The absolute distance of each dog’s measurements from the ideal measurement for each parameter is added together to produce the FRK Gait Inflammation Index Score. This overall score reflects generalized inflammation that impacts mobility resulting from exercise or aging.

### Subjective mobility assessments

The canine brief pain inventory (CBPI) and Liverpool osteoarthritis in dogs (LOAD) questionnaires were completed by kennel staff at the start of the study (d0), before the first and final runs, and 28 h post-first and post-final runs during gait assessment. These questionnaires aimed to evaluate a dog’s pain and mobility changes in response to the exercise regimen. Each timepoint had 3 independent reviewers completing the questionnaire. Due to staffing limitations at the kennel, independent assessors were not the same at each time point.

### Skin and coat assessments

Visual skin and coat assessments were also performed at baseline (d0), mid-study (d43), and end of the study (d74). Three independent reviewers partook in the evaluations, of which 2 of the 3 reviewers were present for all timepoints. One reviewer participated in the first and last assessments, with a fourth independent reviewer filling in for the mid-study timepoint. Assessors graded the severity of alopecia, glossiness, greasiness, softness, and overall quality score on a 5-point scale as previously described (Devriendt et al., 2021), except with overall coat quality ranging from great (1) to very poor (5). The scale used for each measure is listed in Supplementary Table S2. Only the dorsal part of the dog from neck to shoulders to sacrum was examined in these assessments.

### Statistical analysis

Statistical analysis was performed in SAS Studio 3.8 (SAS Institute, Cary, NC). Food consumption data was analyzed as a proportion of food consumed to food offered and in total grams consumed. For both food consumption and fecal quality data, measurements and scores were averaged by week for statistical analysis. The results from the skin and coat assessment as well as the CBPI and LOAD questionnaires were evaluated using a Kruskal-Wallis ANOVA to determine significant differences over time and among treatments at each time point with a pairwise two-sided Dwass, Steel, Critchlow-Fligner (DSCF) multiple comparison post-hoc analysis.

Food consumption, fecal scores, body weights, BCS, activity, and average moving speed data were analyzed using a repeated measures mixed model with fixed effects of sex, time, treatment, treatment × time, and sex × treatment interactions and Tukey-Kramer post-hoc adjustment. Dog within treatment was the repeated subject. Non-significant sex × treatment interactions were removed from the model. Gait parameters and plasma cytokines were analyzed using a repeated measures mixed effects model with fixed effects of timepoint, treatment, and treatment × timepoint interaction and sex as a random variable. For cytokine analysis only, baseline measures were included as a covariate and models were run using type I sum of squares. Significant results were followed with Tukey-Kramer post-hoc adjustment. Data were given Box-Cox transformations when appropriate to meet the normality of residuals assumption. Covariance structure selection was based on variance and covariances of the unstructured model and the lowest corrected Akaike information criterion (AICc). Significance was set at *P *< 0.05 and tendencies were set at 0.05 < *P *≤ 0.08. Data are presented as least square means with their standard errors (SEM). Transformed data are presented as back transformed least square means with standard errors estimated according to Jorgensen and Pedersen (Jorgensen and Pedersen, 1998).

## Results

### Food consumption

No sex × treatment interactions (*P *≥ 0.53) on the percentage or grams of food consumed were observed. Males consumed more grams of food on average than females (*P *= 0.02), but there were no differences between sex in the percentage of food consumed from that offered (*P *= 0.15). Food consumption in grams and percentage of food offered increased steadily over time (*P *< 0.01, Supplementary Figure S1A) but did not differ by treatment or treatment × week interaction (*P *≥ 0.14, Supplementary Figure S1B).

### Fecal quality scores

There were no effects of sex, sex × treatment, treatment, or treatment × time interaction (*P *≥ 0.24) on fecal scores. Fecal quality scores decreased slightly by the end of the study (*P *< 0.01, Supplementary Figure S2), though they did not decrease by more than half a point.

### Body weights and condition

Males weighed significantly more than females, as expected (29.2 ± 0.59 kg vs 26.4 ± 0.59 kg, *P *< 0.01), though females tended to have greater BCS (*P *= 0.07). There were no effects of treatment, sex × treatment, or treatment × time interactions on BW or BCS (*P *≥ 0.25). Body weight declined slightly throughout the study (*P *< 0.01, Supplementary Figure S3A). It was not until week 8 that BW significantly differed from week 1. Dogs lost on average 0.9 kg, or about 3% of their initial body weight, by the end of the study. In agreement with the body weight data, there was a decrease in BCS during the study (*P *< 0.01, Supplementary Figure S3B). However, both treatment averages stayed within the ideal range (4–5 of a 9-point scale) for canine BCS.

### Skin and coat scores

There were no differences between treatments for any of the visual skin and coat parameters at any time point (*P *≥ 0.09, Supplementary Table S3). Greasiness, softness, or overall coat quality scores did not differ among the three timepoints (*P *≥ 0.21). There was a tendency for a decrease in alopecia score in both treatments (*P *= 0.07), where the midway and final timepoints tended to differ, but neither differed from baseline. Glossiness score differed by week (*P *= 0.05), but DSCF post-hoc revealed only tendencies for dogs to have glossier coats at the end of the study (*P *= 0.06). Scaliness scores decreased from the first timepoint (*P *= 0.02).

### Activity

**Figure 1.**
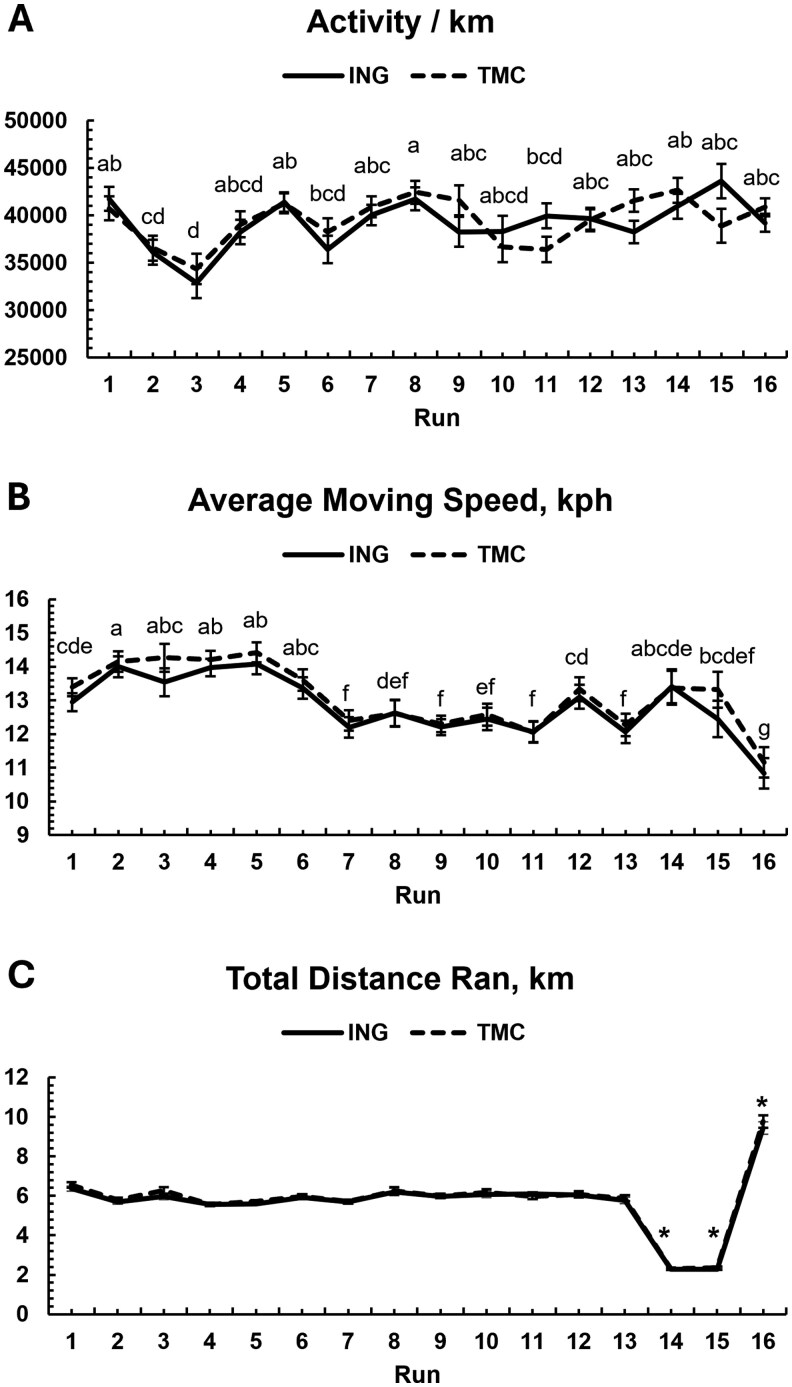
Activity per km by treatment for each run of the study (A), Average moving speed in km per hour for each run of the study (B), and Total distance ran in km for each run of the study (C). Dogs were fed diets with either inorganic (ING) or amino acid complexed (TMC) trace mineral sources. Differing superscripts denote significant differences between weeks (*P *< 0.05).

Treatment did not affect the total activity per km (*P *= 0.70, Figure 1A) or average moving speed (*P *= 0.52, Figure 1B). Activity fluctuated by run (*P *< 0.01), with a dip during run #3, but otherwise, there were no major trends throughout the exercise regimen. There was a significant treatment × time interaction (*P *= 0.05, Figure 1C); however, the ING group did not significantly differ from the TMC group at any run as determined by Tukey’s post-hoc analysis (*P *≥ 0.98). Females exerted more activity on the runs, as registered by the accelerometers, compared to males (40,879 ± 514.31 Actical units/km vs. 37,762 ± 505.15 Actical units/km, *P *< 0.01).

Average moving speed significantly fluctuated by run (*P *< 0.01), where dogs ran faster in the first month of the exercise regimen, but then began to decrease in speed as the regimen progressed. There were no effects of treatment or treatment × time interaction (*P *≥ 0.25). Males had a faster average moving speed compared to females (13.56 ± 0.21 kmph vs 12.36 ± 0.21 kmph, *P *< 0.01).

Distance run in km differed by week (*P *< 0.01), as was designed, with consistent distances covered in runs 1 to 13, decreased distance on runs 14 and 15 of about 2.3 km, then close to 9.5 km run in the final challenge run. There were no differences in distance run between sexes, treatments, or treatment × run interaction (*P *≥ 0.31). There were no treatment × sex interactions for average moving speed, activity per km, or actual distance run (*P *≥ 0.26).

### Visual pain assessments

All aspects of the questionnaires were very low scoring and indicated that dogs were healthy and not experiencing mobility or pain issues as perceived by the handlers. There were no differences between treatments at any timepoint for LOAD questionnaire items concerning the dog’s overall mobility (question 1), how disabled they were by lameness (question 2), their keenness to exercise (question 7), or stiffness in laying down after exercise (question 2, *P *≥ 0.16, Table 2). There were no differences between treatments in CBPI questionnaire items at any timepoint (*P *≥ 0.29, Table 3), except for the final question (*P *< 0.01).

**Table 2.** Mean results from the Liverpool osteoarthritis in dogs (LOAD) questionnaire by treatment at each timepoint

Question	Timepoint	Treatment		*P-*value
		ING	TMC	
**1. How is the dog’s mobility in general?** **(1 – very good, 5 - very poor)**	Baseline	1.13	1.08	0.45
	Pre-initial run	1.02	1.00	0.32
	Post-initial run	1.00	1.00	1.00
	Pre-final run	1.07	1.01	0.16
	Post-final run	1.43	1.30	0.35
**2. How disabled is the dog by their lameness?** **(1 – not at all, 5 - extremely)**	Baseline	1.00	1.02	0.32
	Pre-initial run	1.00	1.00	1.00
	Post-initial run	1.02	1.00	0.32
	Pre-final run	1.02	1.00	0.32
	Post-final run	1.08	1.05	0.63
**3. How active is the dog?** **(1 – extremely, 5 – not at all)**	Baseline	1.33	1.23	0.45
	Pre-initial run	1.05	1.02	0.30
	Post-initial run	1.12	1.07	0.43
	Pre-final run	1.67	1.60	0.57
	Post-final run	1.92	1.65	0.07
**4. What is the effect of cold, damp, weather on the dog’s lameness?** **(1 – none, 5 – extreme)**	Baseline	1.03	1.02	0.55
	Pre-initial run	1.00	1.05	0.08
	Post-initial run	1.12	1.05	0.15
	Pre-final run	1.53	1.47	0.21
	Post-final run	1.20	1.10	0.11
**5. To what degree does the dog show stiffness after laying down?** **(1 – none, 5 – extreme)**	Baseline	1.08	1.00	0.04
	Pre-initial run	1.02	1.13	0.07
	Post-initial run	1.07	1.02	0.16
	Pre-final run	1.02	1.02	1.00
	Post-final run	1.20	1.15	0.30
**6. At exercise, how active is the dog?** **(1 – extremely, 5 – not at all)**	Baseline	1.32	1.28	0.74
	Pre-initial run	1.12	1.17	0.48
	Post-initial run	1.10	1.03	0.12
	Pre-final run	1.77	1.73	0.78
	Post-final run	1.95	1.72	0.07
**7. How keen to exercise is the dog?** **(1 – extremely, 5 – not at all)**	Baseline	1.28	1.23	0.56
	Pre-initial run	1.17	1.15	0.97
	Post-initial run	1.07	1.03	0.19
	Pre-final run	1.78	1.77	0.92
	Post-final run	1.90	1.83	0.55
**8. How would you rate the dog’s ability to exercise?** **(1 – very good, 5 - very poor)**	Baseline	1.23	1.18	0.56
	Pre-initial run	1.08	1.05	0.64
	Post-initial run	1.05	1.00	0.08
	Pre-final run	1.10	1.03	0.20
	Post-final run	1.48	1.35	0.30
**9. What overall effect does exercise have on the dog’s lameness?** **(1 – none, 5 – extreme)**	Baseline	1.57	1.65	0.24
	Pre-initial run	1.07	1.00	0.08
	Post-initial run	1.00	1.00	1.00
	Pre-final run	1.60	1.50	0.30
	Post-final run	1.20	1.20	0.72
**10. How often does the dog rest (stop/sit down) during exercise?** **(1 – never, 5 – very frequently)**	Baseline	2.28	2.27	0.96
	Pre-initial run	1.17	1.03	0.03
	Post-initial run	1.00	1.00	1.00
	Pre-final run	1.60	1.53	0.51
	Post-final run	1.82	1.75	0.51
**11. What is the effect of cold, damp weather on the dog’s ability to exercise?** **(1 – none, 5 – extreme)**	Baseline	1.08	1.03	0.36
	Pre-initial run	1.00	1.03	0.15
	Post-initial run	1.07	1.02	0.16
	Pre-final run	1.18	1.07	0.08
	Post-final run	1.18	1.17	0.68
**12. To what degree does the dog show stiffness in the affected joint after a “lie down” following exercise?** **(1 – none, 5 – extreme)**	Baseline	1.08	1.03	0.60
	Pre-initial run	1.00	1.02	0.32
	Post-initial run	1.00	1.00	1.00
	Pre-final run	1.03	1.00	0.15
	Post-final run	1.20	1.10	0.15
**13. What is the effect of the dog’s lameness on their ability to exercise?** **(1 – none, 5 – extreme)**	Baseline	1.07	1.02	0.29
	Pre-initial run	1.00	1.00	1.00
	Post-initial run	1.00	1.00	1.00
	Pre-final run	1.12	1.07	0.29
	Post-final run	1.18	1.05	0.06

**Table 3.** Mean results from the canine brief pain inventory index (CBPI) questionnaire by treatment at each timepoint

Question	Timepoint	Treatment		*P-*value
		ING	TMC	
**1. Rate the dog’s pain at its worst in the last 14 d (for baseline measure) or 24** h **(for pre/post run measure).** **(0 – no pain, 10 – extreme pain)**	Baseline	0.13	0.07	0.93
	Pre-initial run	0.00	0.00	1.00
	Post-initial run	0.00	0.00	1.00
	Pre-final run	0.05	0.00	0.32
	Post-final run	0.25	0.17	0.43
**2. Rate the dog’s pain at its least in the last 14 d (for baseline measure) or 24** h **(for pre/post run measure).** **(0 – no pain, 10 – extreme pain)**	Baseline	0.05	0.02	0.53
	Pre-initial run	0.00	0.00	1.00
	Post-initial run	0.00	0.00	1.00
	Pre-final run	0.00	0.00	1.00
	Post-final run	0.08	0.07	0.67
**3. Rate the dog’s pain at its average in the last 14 d (for baseline measure) or 24** h **(for pre/post run measure).** **(0 – no pain, 10 – extreme pain)**	Baseline	0.07	0.02	0.29
	Pre-initial run	0.00	0.00	1.00
	Post-initial run	0.00	0.00	1.00
	Pre-final run	0.00	0.00	1.00
	Post-final run	0.15	0.08	0.41
**4. Rate the dog’s pain as it is right now** **(0 – no pain, 10 – extreme pain)**	Baseline	0.05	0.02	0.30
	Pre-initial run	0.00	0.00	1.00
	Post-initial run	0.00	0.00	1.00
	Pre-final run	0.00	0.00	1.00
	Post-final run	0.02	0.00	0.32
**5. How has pain interfered with general activity** **(0 - does not interfere, 10 – completely interferes)**	Baseline	0.07	0.02	0.29
	Pre-initial run	0.00	0.00	1.00
	Post-initial run	0.00	0.00	1.00
	Pre-final run	0.00	0.00	1.00
	Post-final run	0.00	0.00	1.00
**6. How has pain interfered with enjoyment of life** **(0 - does not interfere, 10 – completely interferes)**	Baseline	0.05	0.02	0.30
	Pre-initial run	0.00	0.00	1.00
	Post-initial run	0.00	0.00	1.00
	Pre-final run	0.00	0.00	1.00
	Post-final run	0.00	0.00	1.00
**7. How has pain interfered with ability to rise from lying down** **(0 - does not interfere, 10 – completely interferes)**	Baseline	0.10	0.03	0.31
	Pre-initial run	0.00	0.00	1.00
	Post-initial run	0.00	0.00	1.00
	Pre-final run	0.00	0.00	1.00
	Post-final run	0.02	0.03	0.97
**8. How has pain interfered with ability to walk** **(0 - does not interfere, 10 – completely interferes)**	Baseline	0.03	0.02	0.55
	Pre-initial run	0.00	0.00	1.00
	Post-initial run	0.00	0.00	1.00
	Pre-final run	0.00	0.00	1.00
	Post-final run	0.00	0.00	1.00
**9. How has pain interfered with ability to run** **(0 - does not interfere, 10 – completely interferes)**	Baseline	0.03	0.02	0.55
	Pre-initial run	0.00	0.00	1.00
	Post-initial run	0.00	0.00	1.00
	Pre-final run	0.00	0.00	1.00
	Post-final run	0.03	0.02	0.55
**10. How has pain interfered with ability to climb** **(0 - does not interfere, 10 – completely interferes)**	Baseline	0.05	0.02	0.53
	Pre-initial run	0.00	0.00	1.00
	Post-initial run	0.00	0.00	1.00
	Pre-final run	0.00	0.00	1.00
	Post-final run	0.03	0.05	0.59
**11. Describe the dog’s overall quality of life over the last 14 d (baseline) or 24 h (pre/post run)** **(1 - poor, 5 – excellent)**	Baseline	4.33	4.33	0.30
	Pre-initial run	4.85	4.87	0.94
	Post-initial run	5.00	5.00	1.00
	Pre-final run	4.55	4.60	0.45
	Post-final run	4.45	4.72	< 0.01

Before treatment acclimation, dogs fed TMC minerals were perceived to show less stiffness after laying down (question 5, *P *= 0.04) but did not differ from dogs fed ING minerals for any other parameters in either questionnaire at baseline (*P *≥ 0.24). After acclimation to diets and before exercise, there was a tendency for the opposite, with ING fed dogs perceived as less stiff after laying down (question 5, *P *= 0.07). However, after the initial exercise there were no longer perceived differences in the dogs’ stiffness after laying down (*P *≥ 0.16). There were perceived differences in how often the dogs rested during exercise before the initial run (question 10, *P *= 0.03) and tendencies for differences in the effect of cold weather on dog’s lameness (question 11, *P *= 0.08) and overall effect of exercise on lameness (question 9, *P *= 0.08) before the initial exercise, but none of these traits persisted after exercise began (*P *≥ 0.11). The only perceived difference between treatments after the initial exercise was a tendency for dogs fed TMC minerals to have improved ability to exercise (question 8, *P *= 0.08). This trend did not continue through the final exercise time points (*P *≥ 0.20).

After the course of the exercise regimen and before the final run, there was a tendency for dogs fed TMC minerals to be perceived as less affected by cold, damp weather on their ability to exercise (question 11, *P *= 0.08). After completion of the final run, dogs fed TMC mineral sources tended to be perceived as more active (question 3, *P *= 0.07), more active at exercise (question 6, *P *= 0.07), and less affected by lameness on their ability to exercise (question 13, *P *= 0.06). In agreement with this, the dogs fed TMC minerals were also perceived as having greater quality of life after the final exercise in the CBPI questionnaire (question 11, *P *< 0.01).

### Gait assessment

The FRK Gait Inflammation Index Score was not affected by treatment or treatment × timepoint interaction (*P *≥ 0.47, Table 4). There was a significant effect of time (*P *< 0.01), where gait score improved from the pre- and post-initial run to pre-final run, and post-final run was intermediate.

**Table 4.** Symmetry ratios and averages for gait analysis parameters measured by the Gait4Dogs system by treatment and timepoint (pre or post run)

Item	Treatment								*P-*value		
	ING				TMC				Trt	Time	Trt × Time
	Initial Run		Final Run		Initial Run		Final Run				
	Pre- run	Post-run	Pre-run	Post-run	Pre- run	Post-run	Pre-run	Post-run			
**FRK gait index score**	57 ± 5.3	49 ± 4.6	43 ± 3.5	48 ± 3.6	51 ± 5.3	51 ± 4.6	38 ± 3.5	44 ± 3.6	0.47	< 0.01	0.61
**Step length F:H^1^**	0.998 ± 0.004	1.000 ± 0.003	1.010 ± 0.002	1.001 ± 0.002	1.004 ± 0.004	1.001 ± 0.003	1.009 ± 0.002	1.006 ± 0.002	0.35	< 0.01	0.56
**Step length L:R^2^**	1.034 ± 0.022	1.024 ± 0.012	1.026 ± 0.010	1.007 ± 0.009	1.003 ± 0.022	1.006 ± 0.012	1.004 ± 0.010	0.995 ± 0.009	0.14	0.16	0.79
**Step length avg, cm**	43.65 ± 1.24	44.37 ± 1.24	43.97 ± 1.24	43.65 ± 1.24	43.14 ± 1.24	44.55 ± 1.24	43.23 ± 1.24	42.87 ± 1.24	0.60	< 0.01	0.34
**Stride length F:H**	0.999 ± 0.004	1.001 ± 0.003	1.010 ± 0.002	1.002 ± 0.002	1.004 ± 0.004	1.001 ± 0.003	1.009 ± 0.002	1.006 ± 0.002	0.47	< 0.01	0.73
**Stride length L:R**	0.999 ± 0.003	1.000 ± 0.001	0.997 ± 0.002	1.000 ± 0.001	0.996 ± 0.003	0.998 ± 0.001	1.000 ± 0.002	0.998 ± 0.001	0.64	0.83	0.21
**Stride length avg, cm**	87.40 ± 2.48	88.78 ± 2.48	88.03 ± 2.48	87.37 ± 2.48	86.38 ± 2.48	89.19 ± 2.48	86.54 ± 2.48	85.52 ± 2.48	0.61	< 0.01	0.32
**Step-stride ratio F:H**	0.999 ± 0.001	0.999 ± 0.001	1.001 ± 0.001	0.999 ± 0.001	1.001 ± 0.001	1.000 ± 0.001	1.000 ± 0.001	1.000 ± 0.001	0.12	0.25	0.44
**Step-stride ratio L:R**	1.035 ± 0.020	1.024 ± 0.012	1.031 ± 0.010	1.008 ± 0.010	1.007 ± 0.020	1.008 ± 0.012	1.004 ± 0.010	0.997 ± 0.010	0.14	0.17	0.58
**Step-stride ratio avg**	49.95 ± 0.03	49.99 ± 0.02	49.96 ± 0.02	49.97 ± 0.02	49.95 ± 0.03	49.95 ± 0.02	49.96 ± 0.02	49.95 ± 0.02	0.42	0.83	0.82
**Ambulation time, sec**	1.93 ± 0.145	1.78 ± 0.145	1.74 ± 0.145	1.82 ± 0.145	1.90 ± 0.145	1.80 ± 0.145	1.90 ± 0.145	1.91 ± 0.145	0.29	0.38	0.62
**Cadence, paw strikes/min**	86.3 ± 1.93	83.7 ± 1.93	82.6 ± 1.93	79.5 ± 1.93	86.0 ± 1.93	86.8 ± 1.93	82.2 ± 1.93	79.9 ± 1.93	0.76	< 0.01	0.52
**Step time F:H**	0.988 ± 0.004	0.998 ± 0.004	0.985 ± 0.004	0.992 ± 0.004	0.989 ± 0.004	0.991 ± 0.004	0.983 ± 0.004	0.994 ± 0.004	0.70	0.02	0.45
**Step time L:R**	0.962 ± 0.009	0.983 ± 0.007	0.975 ± 0.006	0.981 ± 0.006	0.992 ± 0.009	0.993 ± 0.007	0.995 ± 0.006	0.991 ± 0.006	0.01	0.36	0.34
**Step time avg, sec**	0.356 ± 0.008	0.365 ± 0.008	0.373 ± 0.008	0.386 ± 0.008	0.354 ± 0.008	0.353 ± 0.008	0.373 ± 0.008	0.381 ± 0.008	0.61	< 0.01	0.74
**Cycle time F:H**	0.988 ± 0.004	0.997 ± 0.004	0.985 ± 0.004	0.991 ± 0.004	0.990 ± 0.004	0.992 ± 0.004	0.983 ± 0.004	0.993 ± 0.004	0.83	0.02	0.60
**Cycle time L:R**	1.000 ± 0.002	0.998 ± 0.002	1.000 ± 0.002	1.002 ± 0.002	1.004 ± 0.002	1.002 ± 0.002	1.004 ± 0.002	1.002 ± 0.002	0.03	0.63	0.74
**Cycle time avg, sec**	0.711 ± 0.017	0.729 ± 0.017	0.747 ± 0.017	0.772 ± 0.017	0.707 ± 0.017	0.706 ± 0.017	0.745 ± 0.017	0.762 ± 0.017	0.61	< 0.01	0.75
**Swing time F:H**	0.970 ± 0.023	0.971 ± 0.016	0.935 ± 0.014	0.953 ± 0.015	0.976 ± 0.023	0.970 ± 0.016	0.933 ± 0.014	0.967 ± 0.015	0.77	< 0.01	0.74
**Swing time L:R**	0.977 ± 0.007	0.991 ± 0.007	0.981 ± 0.007	0.990 ± 0.007	0.988 ± 0.007	0.993 ± 0.007	1.002 ± 0.007	0.990 ± 0.007	0.22	0.24	0.15
**Swing time avg, sec**	0.283 ± 0.006	0.289 ± 0.006	0.298 ± 0.006	0.305 ± 0.006	0.280 ± 0.006	0.289 ± 0.006	0.294 ± 0.006	0.299 ± 0.006	0.59	< 0.01	0.97
**Swing % of cycle F:H**	0.982 ± 0.021	0.974 ± 0.012	0.949 ± 0.010	0.962 ± 0.013	0.986 ± 0.021	0.978 ± 0.012	0.950 ± 0.010	0.974 ± 0.013	0.72	< 0.01	0.85
**Swing % of cycle L:R**	0.978 ± 0.007	0.993 ± 0.007	0.981 ± 0.006	0.989 ± 0.006	0.984 ± 0.007	0.992 ± 0.007	0.998 ± 0.006	0.988 ± 0.006	0.44	0.10	0.14
**Swing % of cycle avg, %**	39.9 ± 0.62	39.8 ± 0.42	40.1 ± 0.40	39.7 ± 0.37	39.7 ± 0.62	40.7 ± 0.42	39.5 ± 0.40	39.4 ± 0.37	0.93	0.11	0.33
**Stance time F:H**	1.002 ± 0.013	1.017 ± 0.009	1.022 ± 0.007	1.019 ± 0.009	1.002 ± 0.013	1.010 ± 0.009	1.018 ± 0.007	1.012 ± 0.009	0.68	0.13	0.97
**Stance time L:R**	1.015 ± 0.005	1.004 ± 0.005	1.014 ± 0.005	1.010 ± 0.005	1.017 ± 0.005	1.009 ± 0.005	1.006 ± 0.005	1.012 ± 0.005	0.99	0.22	0.52
**Stance time avg, sec**	0.428 ± 0.012	0.440 ± 0.012	0.449 ± 0.012	0.468 ± 0.012	0.427 ± 0.012	0.420 ± 0.012	0.451 ± 0.012	0.462 ± 0.012	0.65	< 0.01	0.59
**Stance % of cycle F:H**	1.014 ± 0.015	1.020 ± 0.009	1.038 ± 0.008	1.029 ± 0.009	1.012 ± 0.015	1.018 ± 0.009	1.036 ± 0.008	1.019 ± 0.009	0.69	< 0.01	0.84
**Stance % of cycle L:R**	1.015 ± 0.005	1.006 ± 0.005	1.014 ± 0.005	1.008 ± 0.005	1.012 ± 0.005	1.007 ± 0.005	1.002 ± 0.005	1.009 ± 0.005	0.48	0.20	0.23
**Stance % of cycle avg, %**	60.1 ± 0.62	60.2 ± 0.42	60.0 ± 0.40	60.3 ± 0.37	60.3 ± 0.62	59.3 ± 0.42	60.5 ± 0.40	60.6 ± 0.37	0.94	0.11	0.33
**Mean pressure F:H**	1.019 ± 0.017	1.028 ± 0.017	1.059 ± 0.017	1.050 ± 0.017	1.034 ± 0.017	1.048 ± 0.017	1.090 ± 0.017	1.062 ± 0.017	0.20	< 0.01	0.80
**Mean pressure L:R**	0.972 ± 0.007	0.973 ± 0.007	0.974 ± 0.007	0.960 ± 0.007	0.965 ± 0.007	0.959 ± 0.007	0.966 ± 0.007	0.982	0.72	0.88	0.02
**Mean pressure avg, arb. unit**	2.50 ± 0.02	2.48 ± 0.02	2.48 ± 0.02	2.42 ± 0.02	2.51 ± 0.02	2.51 ± 0.02	2.48 ± 0.02	2.43 ± 0.02	0.62	< 0.01	0.54
**Total scale pressure F:H**	1.164 ± 0.034	1.206 ± 0.034	1.288 ± 0.034	1.230 ± 0.034	1.167 ± 0.034	1.214 ± 0.034	1.280 ± 0.034	1.223 ± 0.034	0.97	< 0.01	0.98
**Total scale pressure L:R**	1.013 ± 0.012	0.987 ± 0.010	0.990 ± 0.009	0.972 ± 0.010	0.990 ± 0.012	0.967 ± 0.010	0.982 ± 0.009	0.987 ± 0.010	0.20	0.01	0.21
**Total scale pressure avg, arb. unit**	48.6 ± 2.99	48.3 ± 3.00	46.9 ± 2.95	45.5 ± 2.95	48.6 ± 2.99	48.7 ± 3.00	46.6 ± 2.95	44.7 ± 2.95	0.92	< 0.01	0.34
**TPI F:H**	1.164 ± 0.034	1.206 ± 0.034	1.288 ± 0.034	1.230 ± 0.034	1.167 ± 0.034	1.214 ± 0.034	1.280 ± 0.034	1.222 ± 0.034	0.97	< 0.01	0.98
**TPI L:R**	1.013 ± 0.012	0.987 ± 0.010	0.990 ± 0.009	0.972 ± 0.010	0.990 ± 0.012	0.670 ± 0.010	0.982 ± 0.009	0.987 ± 0.010	0.21	0.01	0.21
**TPI avg, %**	25.0 ± 0.01	25.0 ± 0.01	25.0 ± 0.01	25.0 ± 0.01	25.0 ± 0.01	25.0 ± 0.01	25.0 ± 0.01	25.0 ± 0.01	0.45	0.46	0.32
**GLS F:H**	0.776 ± 0.023	0.804 ± 0.023	0.859 ± 0.023	0.820 ± 0.023	0.778 ± 0.023	0.810 ± 0.023	0.854 ± 0.023	0.816 ± 0.023	0.98	< 0.01	0.98
**GLS L:R**	1.012 ± 0.009	0.989 ± 0.009	0.990 ± 0.009	0.971 ± 0.009	0.991 ± 0.009	0.968 ± 0.009	0.981 ± 0.009	0.986 ± 0.009	0.24	0.02	0.18
**GLS avg, arb. unit**	102.8 ± 0.30	102.4 ± 0.30	101.7 ± 0.30	102.1 ± 0.30	102.6 ± 0.30	102.3 ± 0.30	101.7 ± 0.30	102.2 ± 0.30	0.89	< 0.01	0.92

TPI: total pressure index; GLS: gait lameness score; F:H: front:hind ratio, L:H: left:right ratio,

1 F:H ratio calculated as the sum of both forelimbs divided by the sum of both hindlimbs

2 L:R ratios calculated as the sum of the left forelimb and left hindlimb divided by the sum of right forelimb and right hindlimb

**Figure 2.**
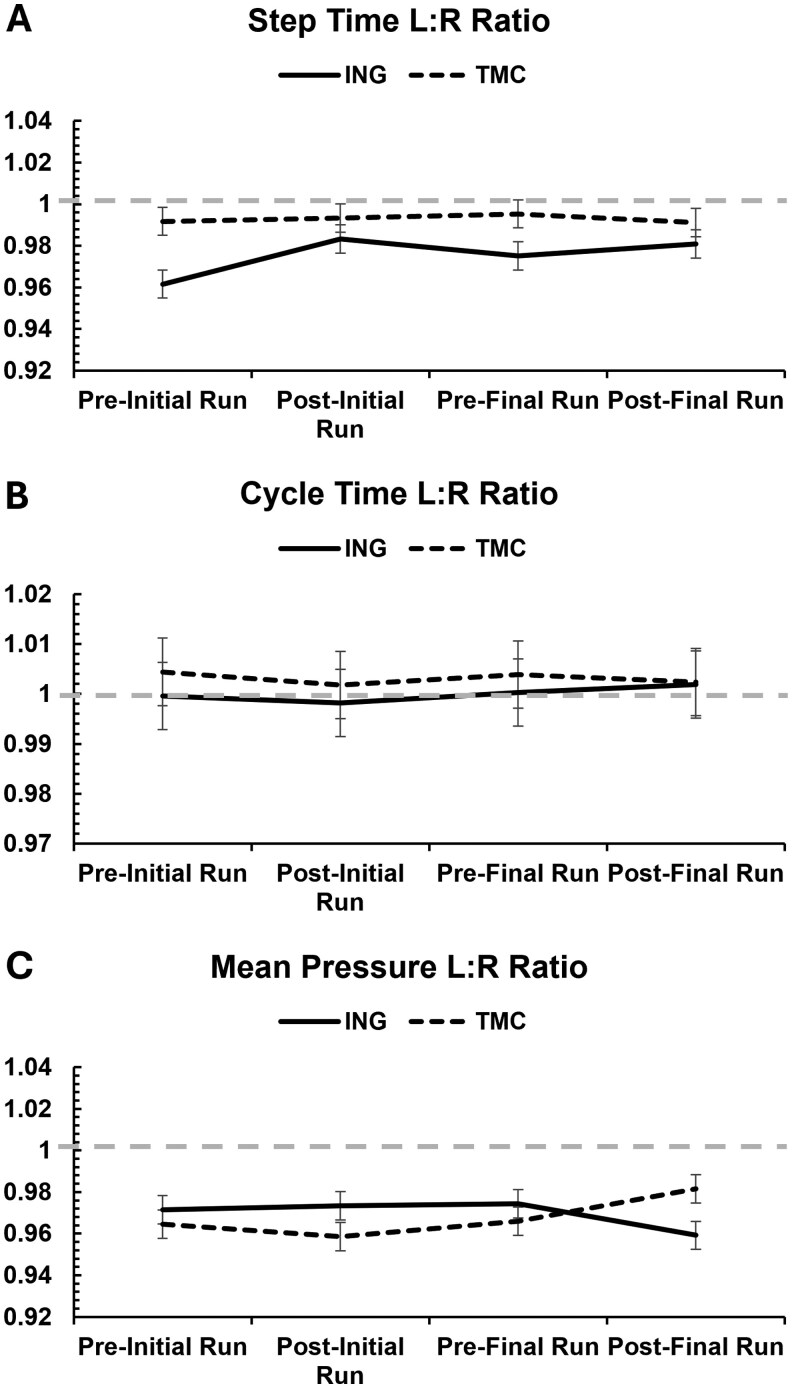
Left:right symmetry ratio for step time (A), cycle time(B), and mean pressure (C) across timepoints during initial and final exercise runs. Dogs were fed diets with either inorganic (ING) or amino acid complexed (TMC) trace mineral sources. Grey dashed line falls along 1:1 ratio.

Step time L:R ratio was significantly greater in dogs fed TMC minerals (*P *= 0.01, Figure 2A), and was closer to 1 indicating a more symmetric step time. Cycle time L:R ratio was also greater in TMC mineral-fed dogs (*P *= 0.03, Figure 2B). While ING fed dogs had a ratio closer to 1, both treatments were extremely close to 1 and overall, very symmetric. There was a significant treatment × timepoint interaction for mean pressure L:R ratio (*P *= 0.02, Figure 2C). Though timepoints did not differ by Tukey’s post-hoc analysis, the L:R ratio in dogs fed TMC minerals increased closer to 1 after the final run while the ratio in dogs fed ING minerals declined further from 1.

Several gait parameters were affected by timepoint. The FRK inflammation index score decreased at the pre-final run timepoint compared to pre- and post-initial run, with post-final run intermediate the two (*P *< 0.01, Table 4). In contrast, the step-length F:H ratio was lowest at the initial run collections, increased by pre-final run, and then was intermediate post-final run (*P *< 0.01). Average step length was increased at the post-initial run, but all other timepoints were similar (*P *< 0.01). The stride length F:H ratio was increased at the pre-final run timepoint compared to both post-initial and post-final run times (*P *< 0.01). Average stride length however was elevated at the post-initial run time point relative to all others (*P *< 0.01). Cadence and average total scaled pressure gradually decreased over time (*P *< 0.01) as average stance time gradually increased (*P *< 0.01). Step-time F:H (*P *= 0.02) and cycle time F:H ratios (*P *= 0.02) were greater at post-initial run compared to pre-final run, with other timepoints intermediate (*P *= 0.02). Average step time (*P *< 0.01), average cycle time (*P *< 0.01), and average swing time (*P *< 0.01) were longer at both final run time points compared to those at the initial run. Swing time F:H ratio (*P *< 0.01) was decreased at the pre-final run compared to all other timepoints (*P *< 0.01). In contrast, total scaled pressure F:H ratio (*P *< 0.01) and TPI % F:H ratio (*P *< 0.01), and GLS F:H ratio (*P *< 0.01) increased at the pre-final run compared to all other timepoints. Swing % F:H ratio was decreased and stance % F:H ratio was increased at pre-final run compared to both post-run timepoints, but pre-initial run was intermediate for both (*P *< 0.01). Mean pressure F:H ratio increased from pre-initial to pre-final run, with both post-run timepoints intermediate (*P *< 0.01). In contrast, average GLS decreased from pre-initial to pre-final run with the post-run timepoints intermediate (*P *< 0.01). Average mean pressure was decreased at the post-final run compared to all other time points (*P *< 0.01). Total scale pressure L:R ratio (*P *< 0.01), TPI% L:R ratio (*P *= 0.01), and GLS L:R ratio (*P *= 0.02) decreased from pre-initial run to pre-final run and both pre- and post-final run were intermediate.

### Circulating cytokines

Results from the cytokine analysis are presented in Table 5. For the analytes IFNγ, TNFα, and IP-10, most samples registered below the detectable range and thus were not analyzed due to insufficient data.

**Table 5.** Plasma cytokine concentrations by run, treatment, and timepoint

ITEM	TREATMENT								*P*-value		
	ING				TMC				Trt	Time	Trt × Time
	Pre- exer.	1 h post	6 h post	24 h post	Pre- exer.	1 h post	6 h post	24 h post			
**GM-CSF, pg/mL**									0.41	< 0.01	0.50
** Initial Run**	26.78 ± 7.94	43.72 ± 19.13	21.15 ± 13.70	35.74 ± 16.20	27.52 ± 8.22	27.15 ± 10.46	14.43 ± 7.67	15.40 ± 5.49			
** Final Run**	30.62 ± 9.42	20.32 ± 7.19	24.41 ± 9.21	27.93 ± 7.02	22.01 ± 5.74	15.91 ± 4.97	26.73 ± 9.62	29.83 ± 7.25			
**IL-2, pg/mL**									0.53	0.40	0.71
** Initial Run**	22.39 ± 6.41	17.94 ± 6.56	12.79 ± 3.07	17.59 ± 7.70	28.53 ± 8.19	22.35 ± 8.44	16.87 ± 3.98	14.61 ± 5.98			
** Final Run**	14.41 ± 6.55	11.53 ± 3.44	13.83 ± 6.07	23.15 ± 7.10	19.78 ± 9.23	17.22 ± 5.59	21.99 ± 10.22	16.15 ± 3.90			
**IL-6, pg/mL**									0.61	0.32	0.83
** Initial Run**	32.61 ± 16.2	34.55 ± 17.59	18.34 ± 9.08	31.09 ± 15.45	29.76 ± 13.34	24.76 ± 11.29	21.92 ± 9.62	17.37 ± 8.32			
** Final Run**	27.28 ± 13.63	24.70 ± 12.67	26.05 ± 13.95	21.34 ± 10.80	16.50 ± 7.24	16.35 ± 7.63	23.37 ± 10.60	14.90 ± 6.65			
**IL-7, pg/mL**									0.80	0.05	0.72
** Initial Run**	23.50 ± 6.18	26.93 ± 7.58	18.52 ± 4.32	16.31 ± 3.57	22.72 ± 5.87	21.33 ± 5.34	24.93 ± 6.75	17.49 ± 3.97			
** Final Run**	26.87 ± 7.55	15.50 ± 3.31	25.18 ± 6.85	20.18 ± 4.92	22.06 ± 5.62	18.69 ± 4.38	27.97 ± 8.02	23.96 ± 6.36			
**IL-8, pg/mL**									0.49	< 0.01	0.68
** Initial Run**	537.43 ± 136.13	747.03 ± 175.18	582.02 ± 93.76	714.44 ± 168.89	414.47 ± 104.99	716.30 ± 167.97	512.14 ± 82.51	606.50 ± 143.38			
** Final Run**	478.09 ± 54.02	551.75 ± 85.69	716.09 ± 100.54	738.19 ± 136.12	478.81 ± 56.64	507.30 ± 78.73	599.80 ± 84.15	749.42 ± 138.19			
**IL-10, pg/mL**									0.07	< 0.01	0.71
** Initial Run**	15.64 ± 2.90	20.91 ± 3.82	15.06 ± 2.99	9.08 ± 1.93	12.06 ± 2.39	18.33 ± 3.27	14.76 ± 2.78	6.10 ± 1.43			
** Final Run**	12.31 ± 2.42	7.99 ± 1.75	15.72 ± 2.91	17.48 ± 3.15	11.57 ± 2.44	8.71 ± 1.87	13.38 ± 2.58	9.22 ± 1.95			
**IL-15, pg/mL**									0.82	0.09	0.89
** Initial Run**	55.85 ± 34.40	58.82 ± 28.09	19.63 ± 13.20	26.06 ± 20.54	31.77 ± 15.55	54.70 ± 23.01	18.89 ± 10.67	14.11 ± 8.75			
** Final Run**	31.26 ± 17.76	29.20 ± 15.79	18.31 ± 10.85	34.51 ± 18.30	27.40 ± 14.51	37.35 ± 19.23	26.42 ± 16.01	24.36 ± 11.06			
**IL-18, pg/mL**									0.87	< 0.01	0.57
** Initial Run**	15.09 ± 4.72	17.68 ± 5.75	9.33 ± 2.59	10.67 ± 3.06	13.34 ± 4.04	11.33 ± 3.30	11.86 ± 3.49	10.02 ± 2.88			
** Final Run**	13.85 ± 4.24	6.20 ± 1.55	13.07 ± 3.95	7.51 ± 1.97	11.58 ± 3.39	7.80 ± 2.07	14.18 ± 4.37	12.17 ± 3.61			
**KC-like, pg/mL**									0.20	< 0.01	0.20
** Initial Run**	12.23 ± 3.58	40.73 ± 8.82	10.49 ± 3.29	11.39 ± 3.39	19.70 ± 5.50	36.85 ± 8.18	21.82 ± 5.92	10.40 ± 3.27			
** Final Run**	7.64 ± 2.51	17.41 ± 4.66	11.79 ± 3.59	11.58 ± 3.69	8.01 ± 2.68	16.22 ± 4.56	19.44 ± 5.23	12.78 ± 3.99			
**MCP-1, pg/mL**									0.09	< 0.01	< 0.01
** Initial Run**	73.75 ± 9.40	96.62 ± 10.65	89.37 ± 9.98	90.50 ± 12.37	94.16 ± 15.32	105.36 ± 12.67	107.46 ± 14.43	77.82 ± 9.14			
** Final Run**	79.30 ± 8.51	78.62 ± 8.57	77.22 ± 7.04	84.96 ± 7.82	78.68 ± 8.38	107.77 ± 16.10	105.06 ± 13.03	98.62 ± 10.54			

**Figure 3.**
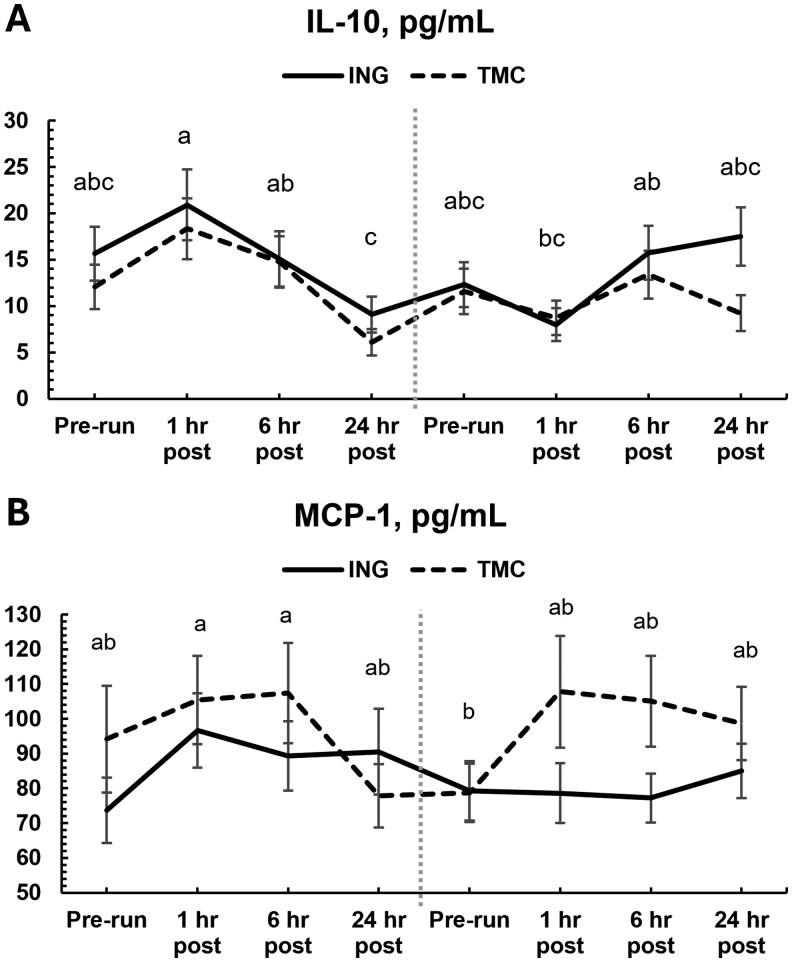
Plasma cytokine concentrations for IL-10 (A) and MCP-1 (B) at pre-exercise, 1 h post-, 6 h post-, and 24 h post-exercise timepoints during initial (left of dashed line) and final (right of dashed line) exercise runs. Dogs were fed diets with either inorganic (ING) or amino acid complexed (TMC) trace mineral sources. Differing superscripts denote significant differences across timepoints (*P *< 0.05).

Circulating IL-10 tended to be lower in dogs fed TMC minerals (*P *= 0.07, Figure 3A). There was also a significant treatment × timepoint interaction for MCP-1 (*P *< 0.01, Figure 3B). While treatments and timepoints did not differ by Tukey’s post-hoc, there was a noticeable elevation in circulating MCP-1 at 1 and 6 h after the final exercise run in dogs fed TMC minerals, while dogs fed ING minerals did not show the same response.

Circulating GM-CSF differed by timepoint after the final run only, where 1-h post-run was significantly lower than 6 and 24 h post-run and baseline was intermediate (*P *< 0.01, Supplementary Figure S4A). Interleukin-8 did not differ among timepoints during the first run but increased from baseline after 6 and 24 h post-run (*P *< 0.01, Supplementary Figure S4B). Interleukin-10 was greater at 1 h post-run than 24 h after the initial run but did not differ among collections during the final run (*P *< 0.01, Supplementary Figure S4C). Circulating IL-18 did not differ among timepoints during the first run but decreased 1 h after the final run compared with baseline and 6 h post run (*P *< 0.01, Supplementary Figure S4D). KC-like protein was elevated 1 h after exercise, then returned to baselines by 6 h at the initial run or remained intermediate baseline and 1-h post exercise after the final run (*P *< 0.01, Supplementary Figure S4E). There was an overall effect of time on MCP-1 concentrations (*P *< 0.01, Figure 3B) where baseline after training was lower than 1 and 6 h post initial run, but there were no significant changes among timepoints within the respective runs. Overall time effect for IL-7 was significant (*P *= 0.05, Supplementary Figure S4F), but timepoints did not differ by post hoc analysis (*P *≥ 0.11). There was no overall effect of time for IL-2, IL-6, or IL-15 (*P *≥ 0.09).

## Discussion

This study examined the effects of a complete and balanced extruded kibble diet formulated with amino acid complexed organic trace minerals, to one containing inorganic trace minerals on mobility, cytokine expression, and skin and coat quality in healthy, sound Labrador Retrievers in a trained and untrained state. These 1:1 metal to amino acid complexed trace minerals are proven to be uniquely absorbed via the amino acid transporters and provide protection from dietary antagonists such as phytic acid, folic acid, and calcium (Gao et al., 2014; Sauer et al., 2017). Dogs fed TMC minerals had modest improvements in gait symmetry and perceived quality of life metrics, particularly after an exercise regimen. Additionally, to the author’s knowledge, this paper is one of the first to report changes in inflammatory cytokines after exercise in a time-course manner during the recovery phase in working Labrador Retrievers.

Overall, trace mineral source did not affect fecal quality scores, food consumption, or body weight. Fecal quality scores were not affected by dietary treatment and only displayed a minor decrease over the course of the exercise regimen. Body weights did decrease throughout the study as dogs underwent the exercise regimen. The weight loss was consistent with increased energy demands from the exercise regimen and remained within the ideal range (4–5 on the 9-point scale) for working dogs. Food refusal was fairly high at the beginning of the trial, which contributed to less than ideal caloric intake to maintain weight during exercise. Diets did not contain extra palatant, unlike typical commercial kibbles, which might have encouraged more food consumption. On average, treatments did not lose more than 5% of their body weight, which has been used previously as a cut-off value in determining maintenance energy requirements via meta-analysis (Divol and Priymenko, 2017). The minor changes to fecal consistency, food consumption, and body weights can be more likely attributed to the stress of the exercise regimen, and overall, the lack of dramatic changes observed in this study support the conclusion that the levels of trace mineral supplementation used in this study were adequate and well tolerated.

Visual skin and coat health were not affected by mineral source in this study. Although slight improvements were noted in alopecia and scaliness over time, the lack of significant differences between treatments suggests that mineral form did not markedly influence skin and coat quality. Some studies have previously demonstrated that dogs fed organic zinc sources (in various forms) can increase zinc deposition into hair (Lowe et al., 1994b; Jamikorn and Preedapattarapong, 2008; Trevizan et al., 2013), possibly contributing to the noted observations in altered hair structure (Jamikorn and Preedapattarapong, 2008; Amundson et al., 2025) which may then produce perceived improvements in texture, color, or glossiness (Trevizan et al., 2013; Pereira et al., 2020). Previous work in senior dogs fed amino acid complexed Zn, Mn, Cu, and Fe had significantly improved hair growth and less shedding compared to those fed sulfate mineral sources (Amundson et al., 2025). The discrepancy between observed effects on skin and coat endpoints in previous studies and the lack of findings in the present study may be due to differences in breeds, ages of the dogs, or environmental exposure. Changes in coat appearance over time in the current study may have been due to weather changes and the cleanliness of the dog. All study dogs were exposed to outdoor elements daily, and rainstorms just before the midpoint and end of study may have given dogs a cleaner, less scaley appearance. Visual coat assessment is used frequently in canine studies (Kirby et al., 2009; Pereira et al., 2020; Devriendt et al., 2021), and although results were consistent between technicians performing the assessment in the present study, it is a subjective measure that can be highly influenced by the reviewers.

Activity levels and average moving speed declined over time, reflecting the cumulative physical demands of the exercise regimen. While treatment effects on these measures were minimal, subtle differences emerged in visual pain and mobility assessments. In this study, we utilized two different questionnaires to assess the perceived pain and mobility of the dogs. The CBPI questionnaire can detect differential improvements in osteoarthritic dogs receiving pain medication vs those receiving placebo (Brown et al., 2008) and has also been validated for use in dogs with bone cancer (Brown et al., 2009). The LOAD questionnaire is considered reliable with acceptable responsiveness and correlates well with the CBPI questionnaire in arthritic dogs (Hercock et al., 2009; Walton et al., 2013). The kennel staff perceived dogs fed TMC minerals as less affected by lameness, cold weather, and exercise-related stiffness at several time points. Additionally, TMC-fed dogs were perceived to have better overall quality of life after the final run, indicating potential long-term benefits of amino acid complexed trace mineral supplementation on subjective measures of well-being.

In this study, there were more tendencies for treatment effect in the LOAD questionnaire compared with the CBPI. This may be because the LOAD questions focus more on mobility and a dog’s mobility during exercise, while the CBPI asks questions that more broadly cover perceived general pain. The highly insignificant results from the CBPI indicate that the exercise regimen did not induce pain in our dogs, but the LOAD results indicated tendencies for altered mobility at various time points. Baseline mobility assessments for the LOAD may have been more variable since dogs are not regularly exposed to the exercise regimen, and so assessment of mobility is based on handler’s memory of dog’s exercise performance, the observed behavior of dogs in the socialization yards, and through daily animal handling. Overall, dogs in this study were perceived as active, very mobile, and without pain, as would be expected for healthy, young adult dogs. As a result, the differences in mobility, while statistically significant, were very small in regard to the assessment scale used. It was likely more difficult to pick up large differences in perceived mobility and pain with a healthy adult population. Another limitation to these questionnaires is that they have been developed for owner assessment of pain and mobility in pet dogs. It is difficult to provide such individualized focus on a dog maintained in a large colony or kennel setting, and as a result, they may not receive as much or as stringent observation as a pet owner would in their own home.

Of the several gait parameters analyzed in this study, only 3 were impacted by treatment. The TMC diet had L:R ratios closer to 1 for step time overall and mean pressure after the final run. The cycle time L:R ratio was extremely close to 1 in both treatment groups. It is not surprising that major findings in the gait data are lacking, as all dogs enrolled in this study were healthy, lean, and young adult dogs. One study assessing gait parameters in healthy vs. osteoarthritic dogs using a different type of pressure walkway system concluded that gait analysis was not suitable for diagnosis of osteoarthritis due to significant overlap of results from healthy and diagnosed patients; however, they noted that the L:R symmetry produced more variability and L:R symmetry of the front legs detected more high-grade lameness (Nielsen et al., 2020). That study also used a mixed variety of breeds, which can all have differing gait characteristics (Carr et al., 2015). Previous research has also found a significant, though weak, correlation between LOAD questionnaire and peak vertical force symmetry index (Walton et al., 2013). Left:right symmetry ratios for gait parameters assessed using the same walkway as was used in this study (GAITRite, CIR Systems) have been previously assessed in healthy Labrador Retrievers and also found 1:1 ratios in healthy subjects (LeQuang et al., 2010). In this study, we also observed some agreement in the sense that the questionnaires and the gait analysis showed the TMC diet appearing slightly more mobile than the ING diet after the final challenge run.

Rather than uncovering subtle changes in gait parameters that may reflect mobility improvements in dogs whose baseline gait is already normal, future studies may clarify whether mineral source confers benefits on gait and mobility in senior dogs, or dogs with diagnosed mobility issues. For example, previous studies in broiler chickens found that supplementation with amino acid complexed minerals yielded a 20% to 25% reduction in lameness due to bacterial chondronecrosis with osteomyelitis (Alrubaye et al., 2020; Alharbi et al., 2024).

This study provides information for an important gap in the literature concerning cytokine concentrations within the recovery phase after exercise in both trained and untrained states. In addition, several of the cytokines/chemokines in this study have not been previously reported for exercise in dogs but demonstrated substantial changes in the recovery period such as KC-like and GM-CSF. Furthermore, studies that have tracked dogs during a long-term exercise regimen did not focus on the immediate recovery phase as done in this study. One study did report acute recovery phase changes in basset hounds during a hunting field trial up to 10 h after the trial, but recorded only IL-1β and TNFα cytokines, for which no changes were observed (Pearson et al., 2020). Contrary to the human literature, we did not observe changes in IL-6 post-exercise. One study reported increased IL-6 in sled dogs during and after endurance exercise (von Pfeil et al., 2015); although other studies found no changes in sled dogs (Wakshlag et al., 2010; Yazwinski et al., 2013; Frye et al., 2018) and previous work in our lab agrees: dogs that underwent a similar exercise regimen had no changes in IL-6 18–20 h post-exercise (Timlin et al., 2024).

Trace minerals like zinc, copper, manganese, iron, and selenium play significant roles in modulating immune responses, including the regulation of monocyte chemotactic protein-1 (MCP-1), a key chemokine involved in recruiting monocytes to sites of inflammation or injury. MCP-1 was the only cytokine changed by treatment in our study. While elevated MCP-1 is frequently observed in incidences of disease or illness in dogs (Duffy et al., 2010; Galán et al., 2018), it has also been shown to play a vital role in muscle regeneration and restoration of function after injury by mediating myoblast proliferation and macrophage infiltration (Warren et al., 2004; Shireman et al., 2007; Lu et al., 2011). Previous studies in exercising dogs found that an intermittent moderate exercise program (Vitger et al., 2017) decreased levels of MCP-1, while sustained strenuous endurance exercise increases MCP-1 (Yazwinski et al., 2013). Concentrations of MCP-1 in our study were well within reported ranges for healthy dogs even with the post-exercise increase (4.2 to 266.8 pg/mL; Duffy et al., 2010), suggesting that we observed a more homeostatic response rather than disease or illness response. Seeing as both treatment groups had very similar average moving speeds, activity per km, and GPS-recorded distances traversed on the final run, it is unlikely that the TMC group exerted themselves more in the final run nor caused more tissue damage than the ING group during exercise. The increased MCP-1 response in dogs fed amino acid complexed trace minerals is likely altering the inflammatory response to exercise and altering acute muscle recovery and regeneration after the final challenge run. This response is consistent with data reported in other species fed amino acid complexed trace minerals during physiologically stressful events in that the complexed trace minerals had a more robust immune response leading to more favorable recovery efficiency (Cheng and Guo, 2004; Pearce et al., 2015; Batistel et al., 2016; Horst et al., 2019).

Tissue repair, including after exercise, involves an intricate and dynamic immune response, which needs to regulate pro- and anti-inflammatory phases. Cross-over studies in humans show MCP-1 is an exercise-induced transient pro-inflammatory signal, with elevated plasma concentrations sustained at least 5 h post-exercise (Wells et al., 2016). Intramuscular MCP-1 is also elevated after exercise and shows a high correlation with increased CD68+ macrophage infiltration 48 h after exercise (Deyhle et al., 2016). In vitro work has also shown that monocytes recruited to injured skeletal muscle, initially with a pro-inflammatory profile, convert to an anti-inflammatory state and convert to macrophages, which then can stimulate myogenesis and muscle fiber growth (Arnold et al., 2007). Deyhle et al.’s (2016) study also showed a reduction in subjective muscle soreness after a second bout of exercise, concurrent with the significant increase in MCP-1 when compared to the first exercise bout. This suggests that increased MCP-1 may be involved in an enhanced recovery resulting in reduced pain after exercise, a finding supported in the current study by the more ideal L:R ratios after the final run and perceived increases in quality of life after exercise recorded in the subjective questionnaires.

A major limitation to this study is that the first and final runs were not of equal distance, thus the impact of a trained state vs untrained state becomes confounded with the impact of the total distance ran. This particular exercise regimen used an 8 km final challenge at the end to ensure dogs would be challenged and show a change in cytokine profiles and mobility, instead of becoming so acclimated that any responses to the running would be dampened. In addition, while the study duration was sufficient to observe acute and subacute responses, it may not have been of sufficient duration to capture long-term effects of supplemental trace mineral source. Future studies should explore the mechanisms underlying observed improvements in gait symmetry and quality of life metrics and assess these diets under varied environmental and workload conditions. The dietary composition should be considered as well with interpretation with these results, particularly in regard to working and sporting dogs. Many athlete and working dogs are likely fed higher-end or specialty diets with more diverse or functional ingredients than used in this study. However, the primary objective of this study was to study the physiological effects of the trace mineral source without potential confounding effects from bioactive or functional ingredients that may be included in a commercial premium diet. It is unknown if inclusion of TMC into those premium diets would add the observed benefits, but addition of TMC may provide an easier means of improving the quality of a given diet compared to complete reformulation.

## Conclusion

This study examined the effects of a trace mineral source (amino acid complexed vs. inorganic) on inflammatory cytokines, gait, and skin and coat quality in adult Labrador Retrievers undergoing a 9-wk exercise regimen. Results reflected very minor changes to gait parameters and MCP-1 concentrations. Minimal differences between treatments were to be expected as we utilized healthy adult dogs that live an active lifestyle. However, improvement at a younger age may set dogs, especially working and military dogs, on a better trajectory for healthy aging. This study was one of the first to track circulating cytokines longitudinally within the first 24 h after exercise in an untrained and trained state in Labrador Retrievers. Amino acid complexed trace minerals, in place of inorganic sources, impacted select areas of perceived and quantified mobility, as well as MCP-1 response to exercise in a trained state.

## Supplementary Material

skaf361_Supplementary_Data

## Abbreviations:

AAFCO: American Association of Feed Control Officials
BCS: body condition score
BW: body weight
CBPI: canine brief pain inventory
F:H ratio: Forelimb: Hindlimb ratio
FRK: Four Rivers Kennel
GM-CSF: granulocyte-macrophage colony-stimulating-factor
GPS: global positioning system
IFNγ: interferon gamma
IL: interleukin
ING: inorganic trace mineral treatment group
IP-10: interferon γ-induced protein 10 kDa
KC-like: keratinocyte chemotactic-like
L:R ratio: Left : Right ratio
LOAD: Liverpool osteoarthritis in dogs
MCP-1: monocyte chemoattractant protein-1
TMC: organic trace mineral complex treatment group
TNFα: tumor necrosis factor alpha
