# Supplementary material for: Gait, skin and coat, and plasma cytokine changes in response to exercise and trace mineral source
Source: J Anim Sci. 2025 Oct 22;104:skaf361. doi: 10.1093/jas/skaf361 (PMC12923159; doi:10.1093/jas/skaf361)

*Supplementary Table 1.* Parameters analyzed by the Gait4Dogs system with their reported units and descriptions.

| **Parameter** | **Unit** | **Description** |
| --- | --- | --- |
| Step length | cm | Measured between the heel center of the current paw print to the heel center of the previous paw print on the opposite foot (i.e.: left front to right front, right  hind to left hind) |
| Stride length | cm | Measured between the heel points of two consecutive paw prints of the same paw (i.e.: left front to left front, right hind to right hind) |
| Step-stride ratio | ratio | Measurement of the ratio between step lengths and stride lengths of the same paw |
| Ambulation time | seconds | the time elapsed between the first contacts of the first and last paw strikes |
| Cadence | Paw strikes per minute | Number of gait cycles per minute as estimated from the first paw contact included in the trial to the last paw strike of the same paw. |
| Step time | seconds | The time elapsed between the first contact time of the previous corresponding limb and the first contact time of the subject limb; with 'previous corresponding limb' defined as the previous limb that matches front vs. hind. |
| Cycle time | seconds | the time elapsed between the first contacts of two  consecutive paw strikes of the same paw (i.e.: left front to left front) |
| Swing time | seconds | The swing phase is the non-weight-bearing portion of each Gait Cycle. It is the time elapsed between the last contact and the first contact of one identified paw |
| Swing % of cycle | percentage | Swing time for each paw presented as percentage of gait cycle for the same paw |
| Stance time | seconds | The stance phase is the weight-bearing portion of each gait cycle. It is the time elapsed between the First Contact and the Last Contact of one identified paw. |
| Stance % of cycle | percentage | Percentage of stance time compared to gait cycle time |
| Mean Pressure | arbitrary unit | Average pressure reading for each paw |
| Total Scale Pressure | arbitrary unit | The sum of peak pressure values recorded from each  activated sensor by a paw during mat contact, represented by the switching levels and reported as a scaled pressure from 0 to 7 for each sensor. (This is related to but not equal to Peak Vertical Force.) |
| Total Pressure Index (TPI) | percentage | The total scaled pressure expressed as a percentage of all four limbs. This shows % of weight distribution across all four paws. |
| Gait4Dogs Lameness Score (GLS) | arbitrary unit | System calculated score based on 100 for each leg. Above 100 indicates compensation of the limb, less than 100 indicates pain during stance |

*Supplementary Table 2.* Description of each parameter used in the skin and coat visual assessment, as well as the corresponding scales used for each parameter.

| **Measure** | **Description** | **Scale** | | | | |
| --- | --- | --- | --- | --- | --- | --- |
| Alopecia | visual assessment on if the dog is experiencing any thinning of coat or baldness | 1  None | 2  Minor | 3  Moderate | 4  Significant | 5  Bald |
| Glossiness | visual assessment of coat’s shine | 1  Highly Reflective | 2 Significantly Reflective | 3  Reflective | 4 Moderately Dull | 5  Very Dull |
| Greasiness | tactile assessment of whether coat has a greasy feeling | 1  Very Dry | 2  Moderately Dry | 3  Normal | 4  Greasy | 5  Very Greasy |
| Softness | tactile assessment of how soft the coat feels | 1  Very Soft | 2  Soft | 3  Moderate | 4  Coarse | 5  Very Coarse |
| Scaliness | assessed between the scapulae, the mid back, and lower back by lifting the hairs and evaluating severity of flakiness/scales | 1  None | 2  Minor | 3  Moderate | 4  Significant | 5  Highly |
| Overall Quality | general overall feel and appearance of coat health | 1  Great | 2  Good | 3  Average | 4  Poor | 5  Terrible |

*Supplementary Table 3.* Average scores for visual skin and coat assessments by treatment and time point.

| **Item** | **Treatment** | | ***P* - Value (Treatment)** |
| --- | --- | --- | --- |
|  | **ING** | **TMC** |  |
| Alopecia |  |  |  |
| Baseline | 1.12 ^xy^ | 1.03 ^xy^ | 0.11 |
| Mid-point | 1.07 ^x^ | 1.08 ^x^ | 0.71 |
| End | 1.03 ^y^ | 1.00 ^y^ | 0.15 |
| Glossiness |  |  |  |
| Baseline | 3.13 ^x^ | 2.85 ^x^ | 0.17 |
| Mid-point | 2.90 ^xy^ | 2.93 ^xy^ | 0.96 |
| End | 2.48 ^y^ | 2.80 ^y^ | 0.16 |
| Greasiness |  |  |  |
| Baseline | 2.78 | 2.92 | 0.22 |
| Mid-point | 2.83 | 2.83 | 0.83 |
| End | 2.98 | 2.90 | 0.40 |
| Softness |  |  |  |
| Baseline | 3.25 | 2.87 | 0.09 |
| Mid-point | 3.00 | 3.02 | 0.93 |
| End | 2.85 | 2.93 | 0.48 |
| Scaliness |  |  |  |
| Baseline | 1.35 ^a^ | 1.27 ^a^ | 0.85 |
| Mid-point | 1.10 ^b^ | 1.13 ^b^ | 0.88 |
| End | 1.15 ^b^ | 1.08 ^b^ | 0.44 |
| Overall Quality |  |  |  |
| Baseline | 3.03 | 2.72 | 0.12 |
| Mid-point | 2.77 | 2.78 | 0.98 |
| End | 2.78 | 2.77 | 0.88 |

^a,b^ superscripts within column denote significant (*P* < 0.05) differences over time.

^x,y^ superscripts within column denote trending (0.05 < *P* ≤ 0.08) differences over time

*Supplementary Figure 1***.** Average weekly feed consumption, displayed as percentage of feed consumed over feed offered, for treatments throughout the duration of the study. Dogs were fed diets with either inorganic (ING) or amino acid complexed (TMC) trace mineral sources. Differing superscripts denote significant differences by week (*P* < 0.05).


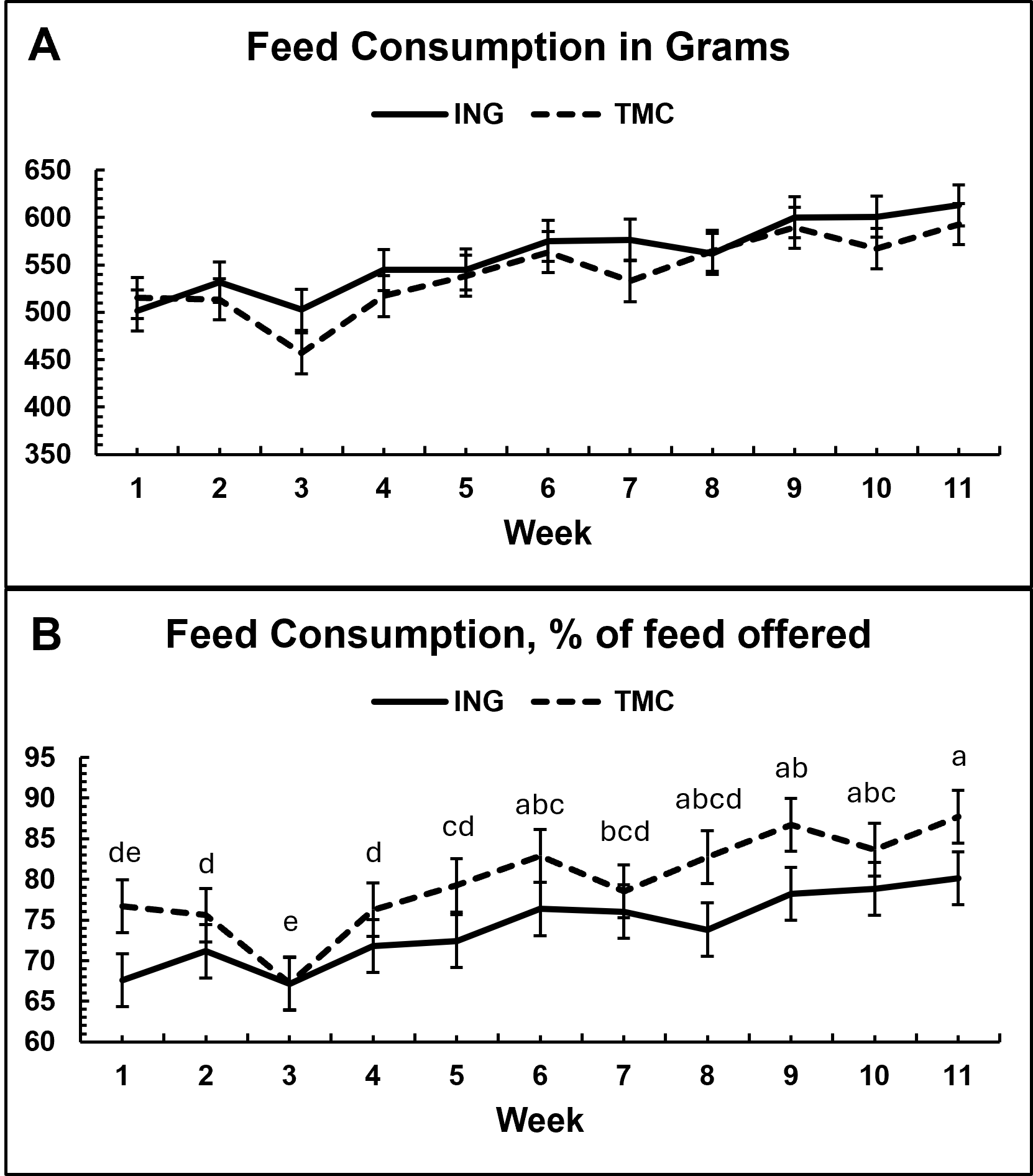


*Supplementary Figure 2***.** Average weekly fecal scores for all treatments throughout the duration of the study. Dogs were fed diets with either inorganic (ING) or amino acid complexed (TMC) trace mineral sources. Differing superscripts denote significant differences by week (*P* < 0.05).

**
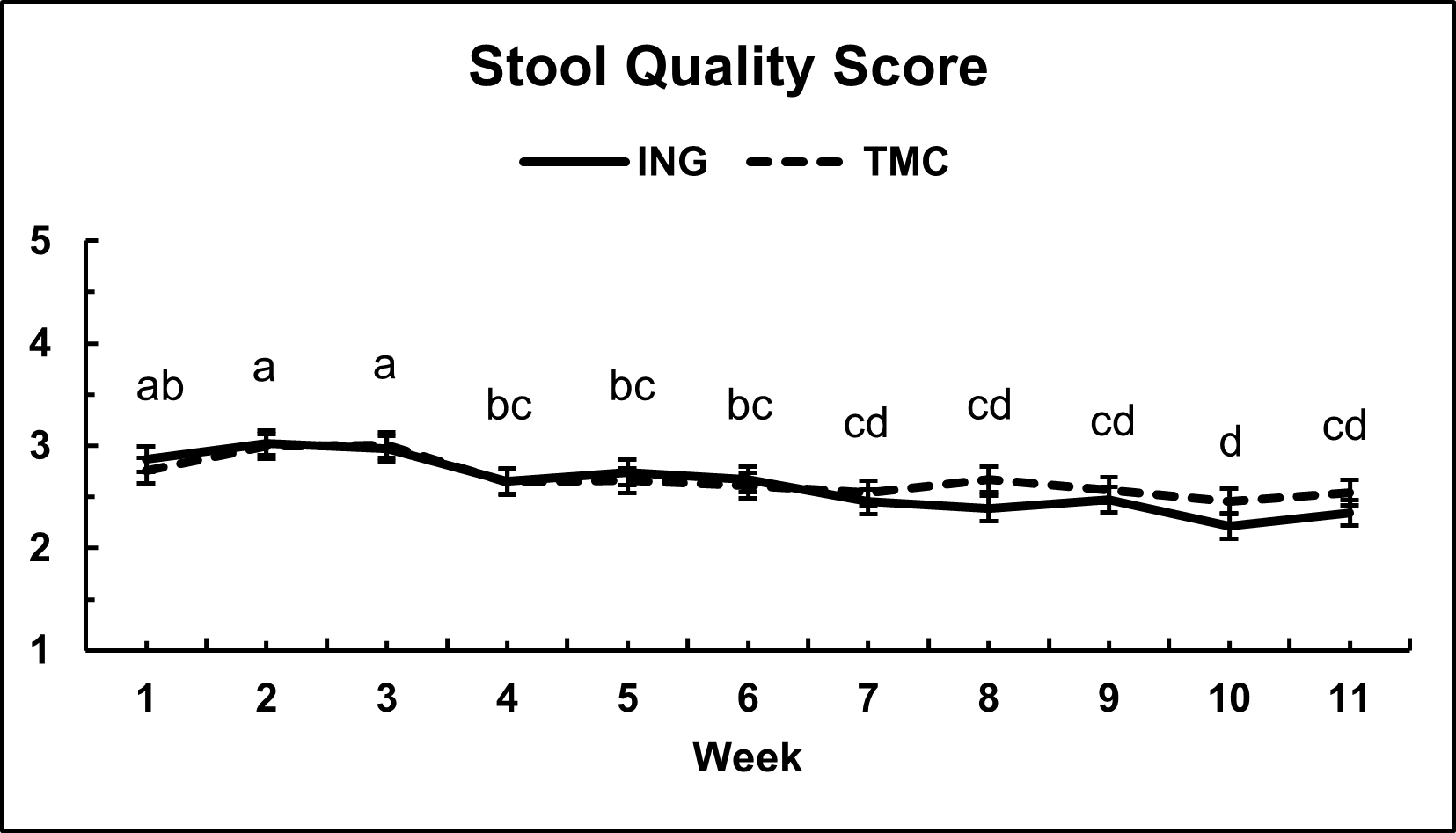
**

*Supplementary Figure 3*. Average body weights in kg (A) and body condition scores (B) by treatment over the course of the study. Condition scores are based on a scale of 1 to 9, with 1 extremely thin and 9 very obese. Ideal score range of 4 to 5. Dogs were fed diets with either inorganic (ING) or amino acid complexed (TMC) trace mineral sources. Differing superscripts denote significant differences between weeks (*P* < 0.05).


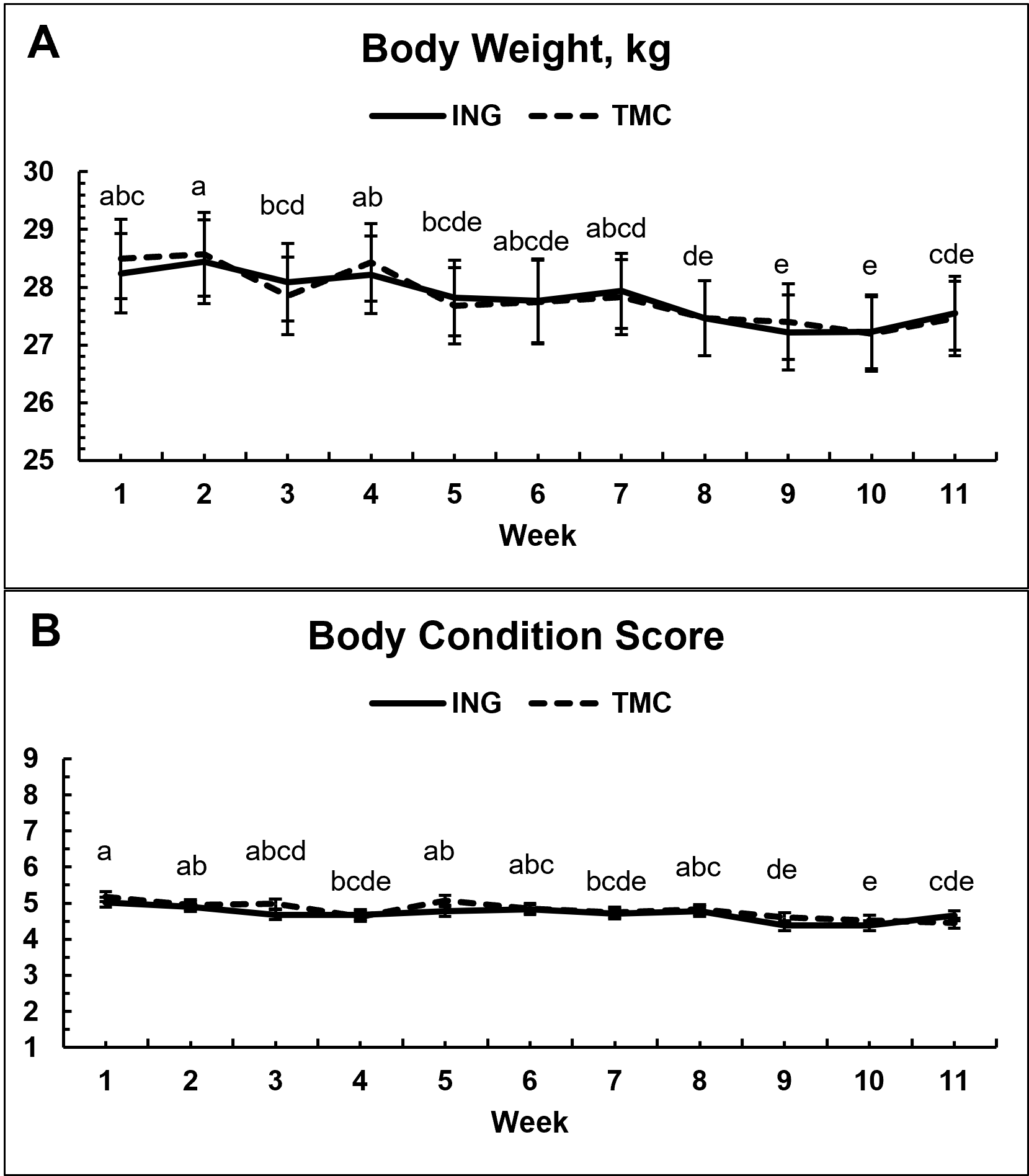


*Supplementary Figure 4*. Changes in plasma cytokine concentrations for GM-CSF, IL-8, IL-10, IL-18, KC-like, and IL-7 in response to the initial (left of dashed line) and final (right of dashed line) exercise runs. Blood was collected pre-run and 1, 6, and 24 hr post-run. Differing superscripts denote significant differences across timepoints (*P* < 0.05).


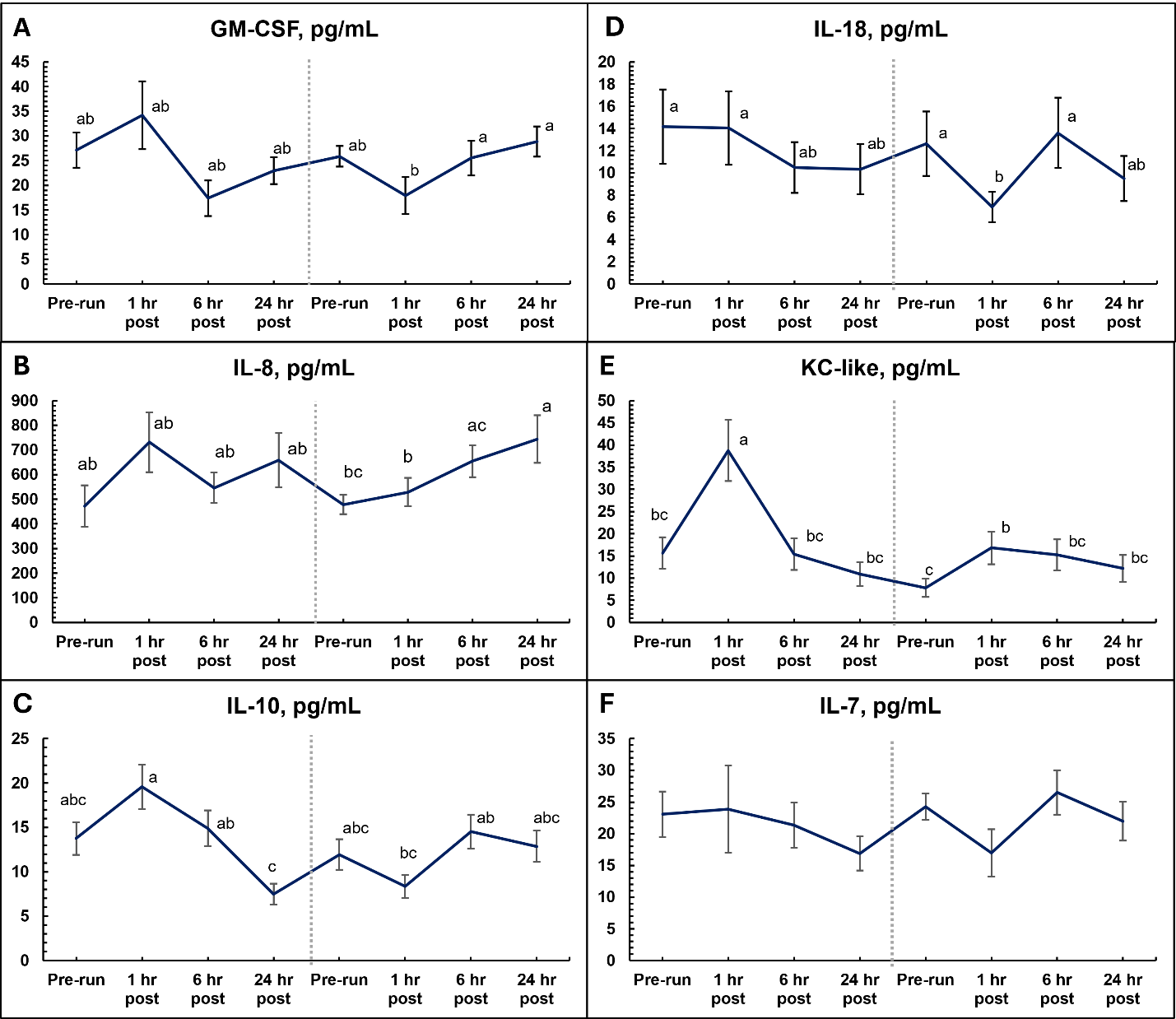

Supplement: skaf361_Supplementary_Data [file skaf361_supplementary_data.zip › ZP JAS Supplementary Materials.docx]
